# Supplementary material for: Human mitochondrial ferritin exhibits highly unusual iron-O2 chemistry distinct from that of cytosolic ferritins
Source: Nat Commun. 2025 May 20;16:4695. doi: 10.1038/s41467-025-59463-1 (PMC12092714; doi:10.1038/s41467-025-59463-1)
Supplement: Supplementary file 1 — Supplementary Information [file 41467_2025_59463_MOESM1_ESM.pdf]

Supplementary Information

Human mitochondrial ferritin exhibits highly unusual iron-O_2 chemistry distinct from that of cytosolic ferritins.

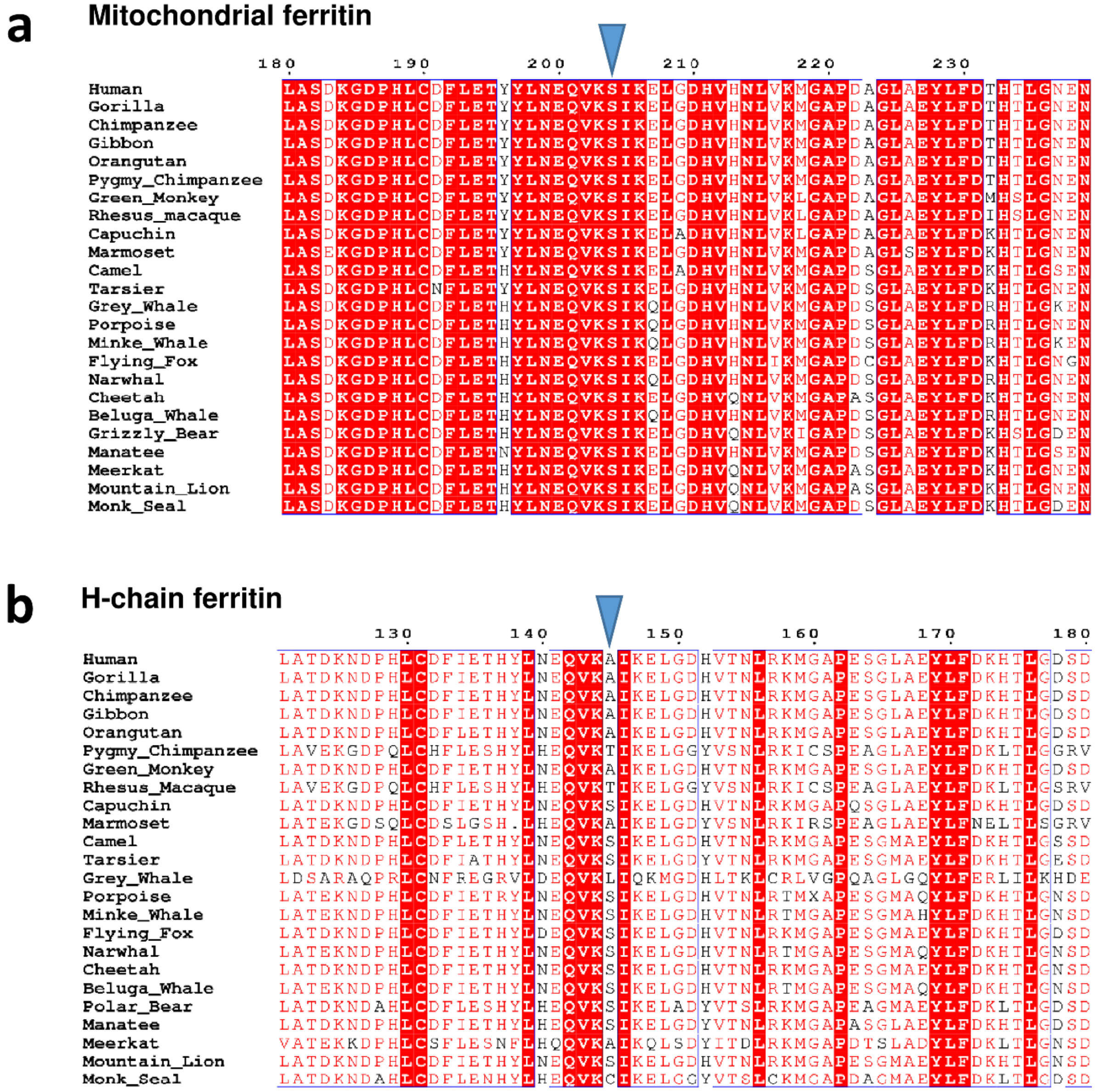

Figure S1. Peptide sequences of (**a**) selected predicted FtMt proteins around sequence position 144 and (**b**) the equivalent sequences of selected cytosolic H-chain ferritins. The blue arrow head indicates the position of a serine (Ser144) that is conserved among mitochondrial ferritins but not their cytosolic equivalents. Note that the numbering for the FtMt sequence refers to the full length protein, including the targeting sequence.

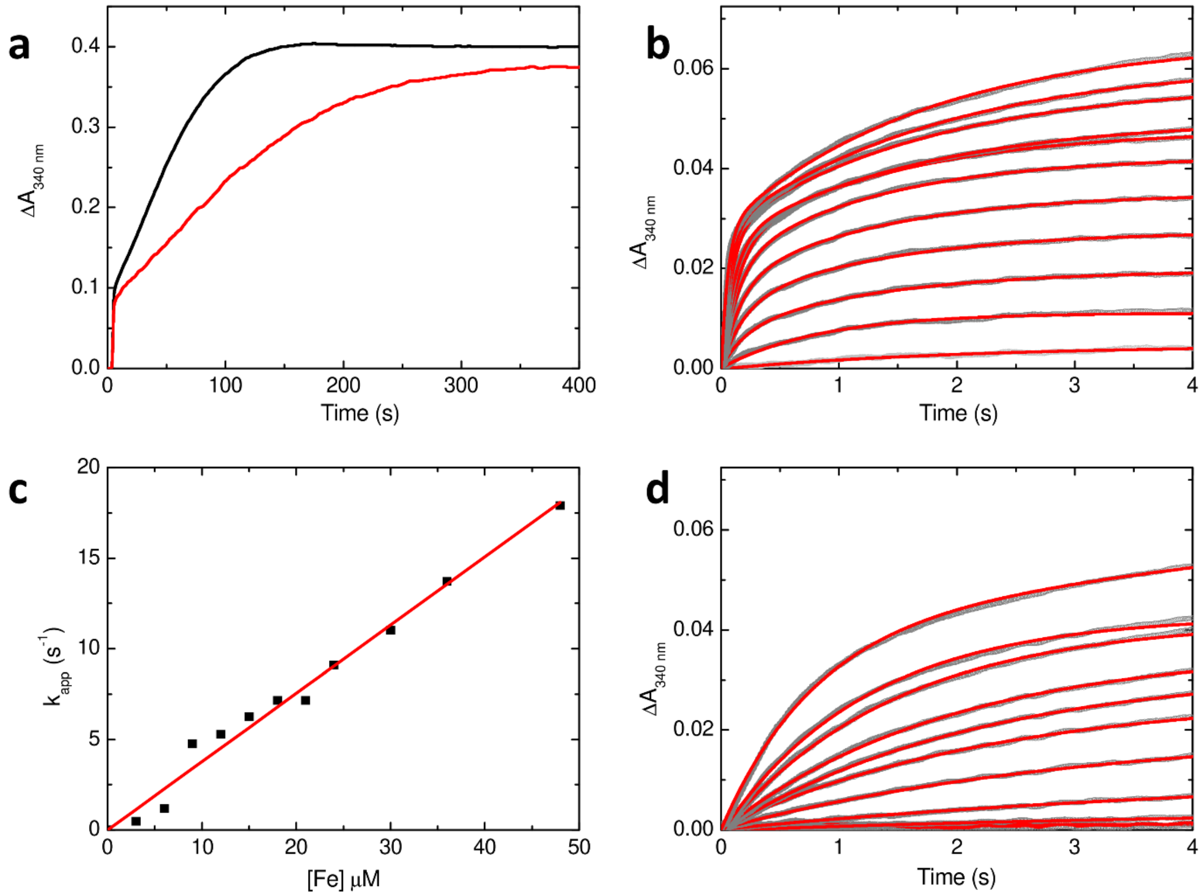

Figure S2. Iron oxidation by wild-type and variant Y34F HuHF. (**a**) Increase in absorbance at 340 nm following the aerobic addition of 400 equivalents of Fe^{2+} to 0.5 µM wild-type (black) or variant Y34F (red) HuHF.(**b**) Equivalent data following aerobic mixing of wild-type HuHF and Fe^{2+} leading to final protein concentration of 0.5 µM and iron concentrations between 3 and 48 µM. Data points represented by open grey circles and fits to mono or biexponential functions (as required) by red traces. (**c**) Dependence of the rapid rate of iron oxidation (as apparent first-order rate constants) extracted from the fits in (b) on the concentration of Fe^{2+}, yielding a second order rate constant of 3.8×10^5 M^{-1} s^{-1}. (**d**) as (b) but for variant Y34F HuHF. Source data are provided as a Source Data file.

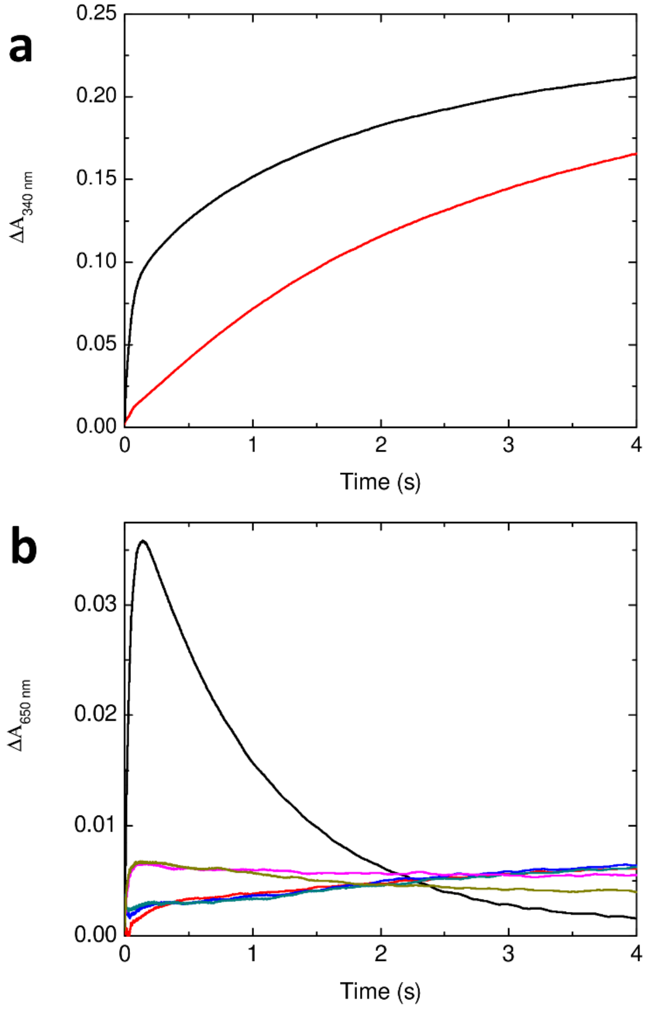

Figure S3. The Y34F substitution slows the rate of Fe^{2+} oxidation at FtMt ferroxidase centres. (**a**) Increase in 340 nm absorbance as a function of time following the mixing of 8.33 μM anaerobic wild-type (black) or variant Y34F (red) FtMt containing 48 Fe^{2+} per cage with an equal volume of buffer containing 250 μM dissolved O_2 at 10 ˚C. (**b**) Absorbance change at 650 nm as a function of time for samples equivalent to those in (a) and variant Y34F at 15 (blue) 20 (teal) 25 (magenta) and 30 (dark yellow) ˚C. Source data are provided as a Source Data file.

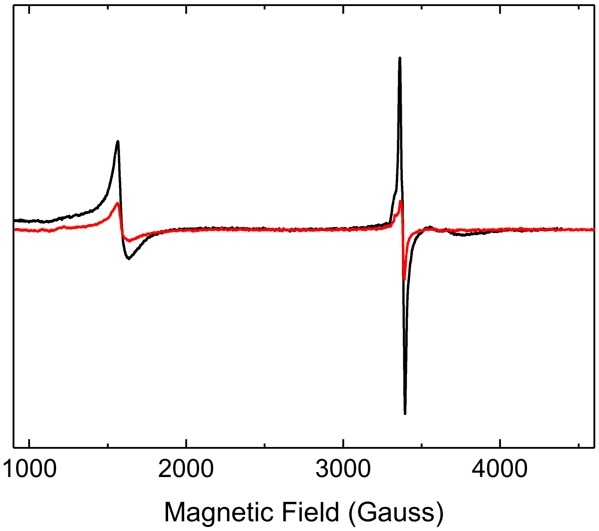

Figure S4. Lack of MVFC formation in the reaction cycle of cytosolic human H-chain ferritin. The EPR spectrum of human H-chain ferritin following anaerobic incubation with 48 equivalents of Fe^{2+} prior to mixing with oxygenated buffer giving final concentrations of 4.17 µM human H-chain ferritin, 200 µM Fe^{2+} and 50 µM O_2 (black trace) and 4.17 µM variant Y34F human H-chain ferritin aerobically mixed with 300 µM Fe^{2+} (red trace). Samples were frozen approximately 10 s after initiating the oxidation reactions. The spectra lack the signals at magnetic fields greater than 3400 Gauss characteristic of MVFC formation, which are evident in equivalently treated samples of mitochondrial ferritin (see Fig. 4 of the main paper). Source data are provided as a Source Data file.

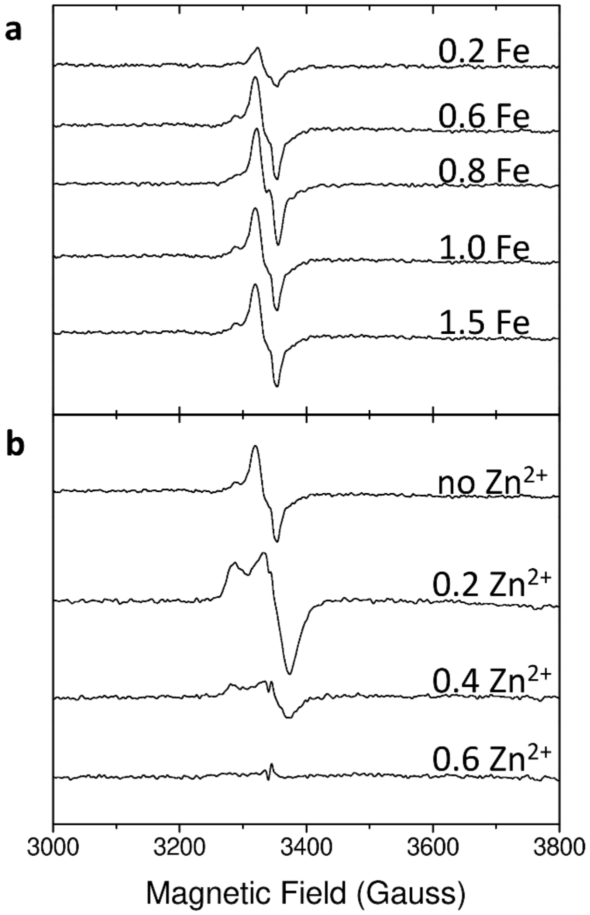

Figure S5. The effect of Fe^{2+}:protein stoichiometry and pre-incubation with Zn^{2+} on Fe^{2+} oxidation catalyzed by FtMt. The dependence of the rate of Fe^{2+} oxidation at the FC of variant Y34F on Fe^{2+} concentration suggested that Fe^{2+} binding to the catalytic site is impaired in this protein (Fig. 2d). This raised the possibility that observation of an MVFC during the Fe^{2+} oxidation was simply due to some FC sites being partially occupied by Fe^{2+}, with subsequent transfer of an electron to a di-Fe^{3+} FC. Alternatively, unreacted Fe^{2+} remaining in solution may be able to displace one Fe^{3+} from FCs (demonstrated to lead to MVFC formation in HuHF [1]. The consequence of partial occupancy of wild-type FtMt FCs was probed by EPR following the aerobic exposure (O_2 in excess) of wild-type FtMt to Fe^{2+} at concentrations below that required to fill all FC sites. (**a**) shows EPR spectra of wild-type FtMt frozen 10 s after aerobic mixing with the indicated stoichiometry of Fe^{2+} per FOC iron-binding site. Additionally, experiments were performed in which 3 equivalents of Fe^{2+} per FC were added following pre-incubation with variable amounts of Zn^{2+}, a potent inhibitor of ferritin activity that acts by binding at FC sites preventing the binding of Fe^{2+} substrate [2]. (**b**) shows EPR spectra of wild-type FtMt pre-incubated with the indicated stoichiometry of Zn^{2+} per FOC iron-binding site prior to aerobic mixing with 1.5 Fe^{2+} per iron-binding site and freezing 10 s after initiating the reaction. Data indicate that catalysis of O_2-driven Fe^{2+} oxidation by FtMt is suppressed at a stoichiometry of 1.2 Zn^{2+} per FOC (0.6 per FOC iron-binding site). Experiments in both (a) and (b) could result in FCs containing only one Fe^{2+} ion, with potential for electron transfer to a di-Fe^{3+} FC. In (b), pre-incubation with Zn^{2+} also results in unreacted Fe^{2+} remaining in solution and able to displace Fe^{3+} from the di-Fe^{3+} sites expected to form in the initial 500 ms of the reaction. However, the resulting spectra contained no evidence of MVFC formation. Source data are provided as a Source Data file.

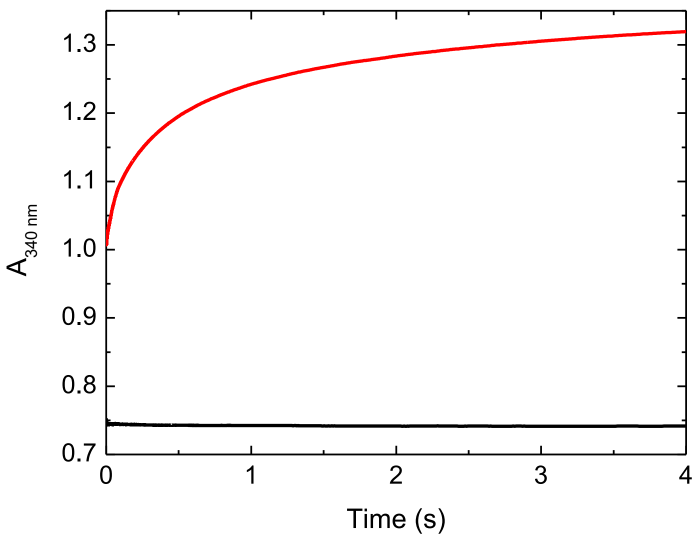

Figure S6. Reaction of FtMt in the MVFC form with O_2. Mixing of 60 μL of a 50 mM Fe^{2+} solution with 3 mL of 20.8 μM wild-type FtMt within a gas-tight Hamilton syringe led to a solution with absorbance at 340 nm of 0.7. Rapid stopped-flow mixing with an equal volume of anaerobic MES pH 6.5 resulted in little change in absorbance (black trace), whilst mixing with aerobic buffer (red trace) resulted in an increase in absorbance to approximately 1.0 within the dead time of the instrument followed by a slower increase to a final value of approximately 1.3. The experiment was performed in triplicate using a single protein preparation. Source data are provided as a Source Data file.

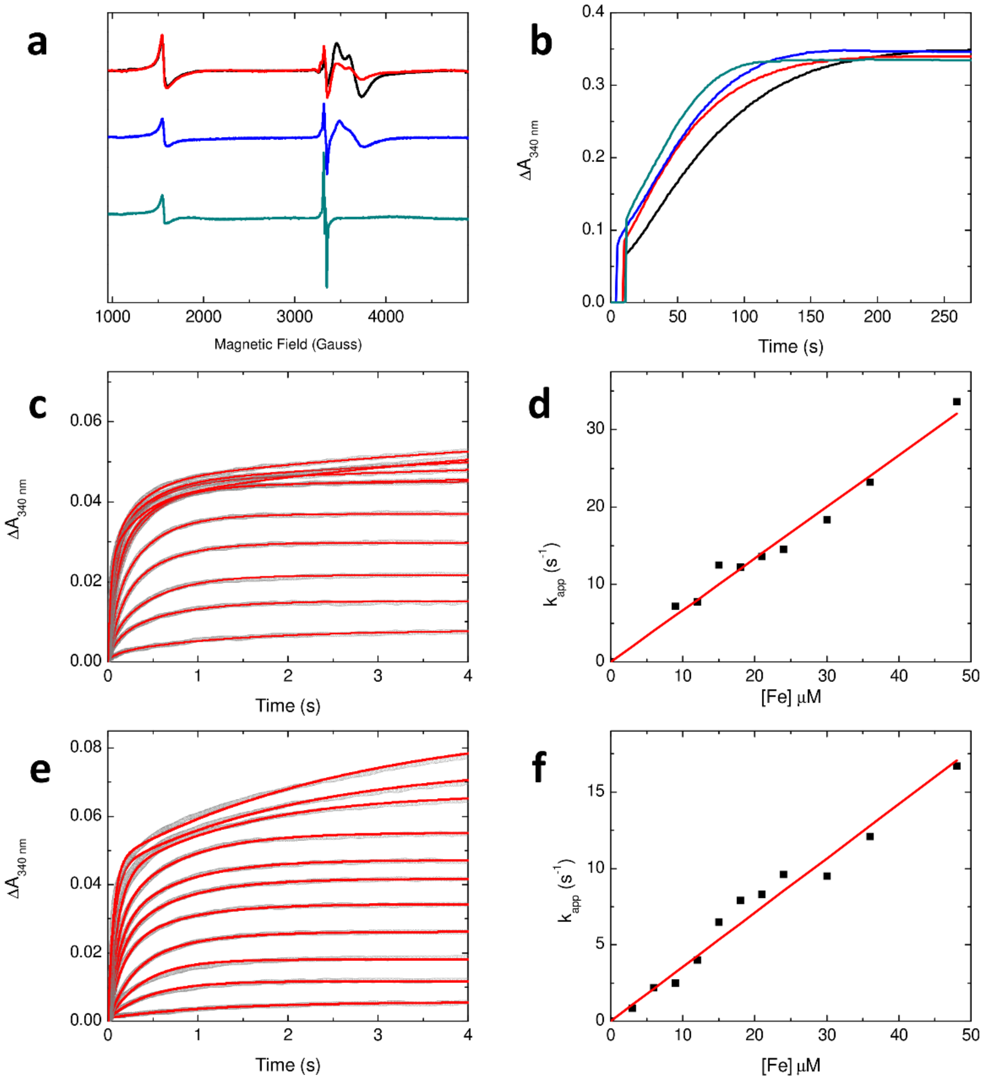

Figure S7. The importance of Ser144 for FtMt activity. (**a**) EPR spectra of the products of FtMt activity under limiting O_2. The EPR spectrum of 300 μL of a 20.8 μM solution of wild-type FtMt frozen 10 s after aerobic mixing with 6 μL of a 50 mM Fe^{2+} solution (50 Fe^{2+} per FtMt, O_2 sufficient to oxidize 50% of added Fe^{2+}, black trace). Red trace shows the equivalent spectrum following the equal volume mixing of 41.6 μM protein and 2 mM Fe^{2+} solutions (50 Fe^{2+} per FtMt, O_2 sufficient to oxidize 50% of added Fe^{2+}). We note that the yield of MVFC for the latter was reduced compared to the addition of a small volume of 50 mM Fe^{2+}. This reduction may have been due to the introduction of further O_2 to the solutions during mixing. Blue and cyan traces are equivalent to the black trace but using FtMt S144A and HuHF A144S in place of wild-type FtMt. (**b**) Mineralization activity of FtMt S144A (red trace) with wild-type protein in black for comparison, and of HuHF variant A144S (blue trace) with wild-type protein in cyan for comparison. (**c**) Increase in 340 nm absorbance following stopped-flow mixing of FtMt S144A with Fe^{2+} solutions of varying concentration to give final concentration of 0.5 μM protein and 3-48 μM Fe^{2+}. Data shown as open grey circles and fits to mono or bi exponential functions (as required) as red traces. (**d**) Dependence of the rapid rate of iron oxidation extracted from the fits in (c) on iron concentration. (**e**) and (**f**) as for (c) and (d) but using HuHF A144S. Experiments with FtMt S144A and HuHF A144S were performed using a single protein preparation in triplicate for absorbance, and together with the appropriate wild-type protein as a control for EPR. Source data are provided as a Source Data file.

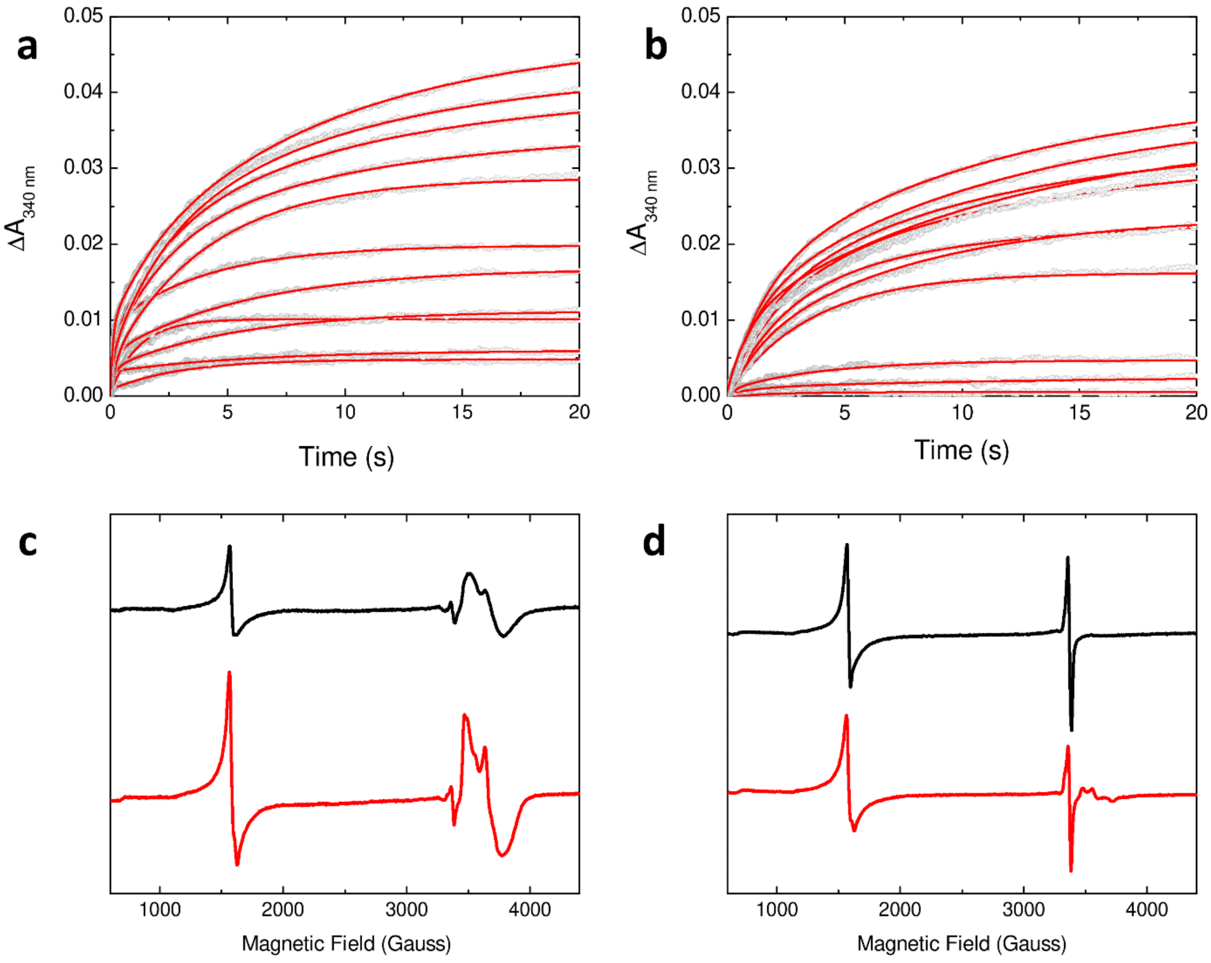

Figure S8. Reactivity of FtMt towards H_2O_2. The increase in 340 nm absorbance as a function of time following the anaerobic equal volume mixing of 1 µM solutions of (**a**) wild-type and (**b**) Y34F FtMt containing 500 µM H_2O_2 with Fe^{2+} solutions of concentration 6-96 µM. Open gray circles indicate data points, red traces represent fits to mono- or bi-exponential fits as required. (**c**) EPR spectra of wild-type (black) and Y34F (red) FtMt anaerobically incubated with 300 µM Fe^{2+} prior to mixing with 100 µM H_2O_2 and frozen approximately 10 s after H_2O_2 addition. (**d**) as (a) but using 500 µM H_2O_2. The data confirm that peroxide driven oxidation of iron by FtMt is much slower than the reaction with oxygen but in both cases variant Y34F forms significant quantities of MVFC whilst the wild type protein only does so when iron is present in excess of oxidant, regardless of co-substrate. Source data are provided as a Source Data file.

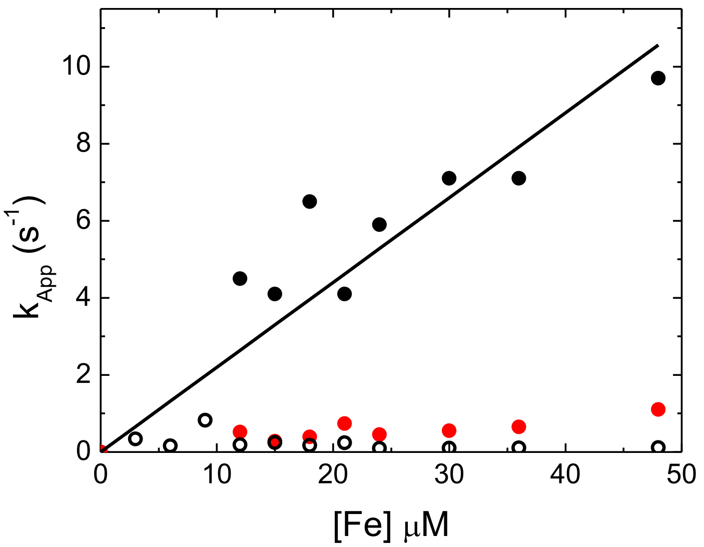

Figure S9. Reaction of FtMt with H_2O_2 is complex. Fitting of kinetic data shown in Fig. S8 required a bi-exponential function for wild-type FtMt. The amplitude of the initial, fast phase was small, accounting for only ~10% of the total amplitude. A plot of the first order rate constant associated with this fast phase is shown (black circles). The small amplitude of this phase resulted in a greater uncertainty in the apparent first order rate constants, but fitting of their dependence to a linear function yielded a second order rate constant of 2.2 $\times 10^5$ M^{-1} s^{-1}. This is similar to that determined from a similar analysis of the 10-fold higher amplitude of the rapid phase observed upon reaction with O_2, which also was Fe^{2+}-dependent. This indicated that the rate-limiting step of the reaction was Fe^{2+} binding. Although it is unclear what the small increase in amplitude observed upon mixing of Fe^{2+} with FtMt in the presence of H_2O_2 is due to, it was also rate-limited by Fe^{2+} binding. Rate constants associated with the second, slower phase are also plotted (black open circles), consistent with a much slower reaction that is independent of Fe^{2+} concentration. A similar analysis of data acquired for Y34F FtMt revealed that there was no fast phase and so a single exponential function was used for fitting. The resulting rate constants are plotted above (red circles), indicating that this slow phase is independent of Fe^{2+} concentration. These data suggest that the conserved Tyr may also be important in activating the FC of FtMt to react with H_2O_2, but the effect of the absence of the Tyr is much less severe than for the reaction with O_2. Source data are provided as a Source Data file.

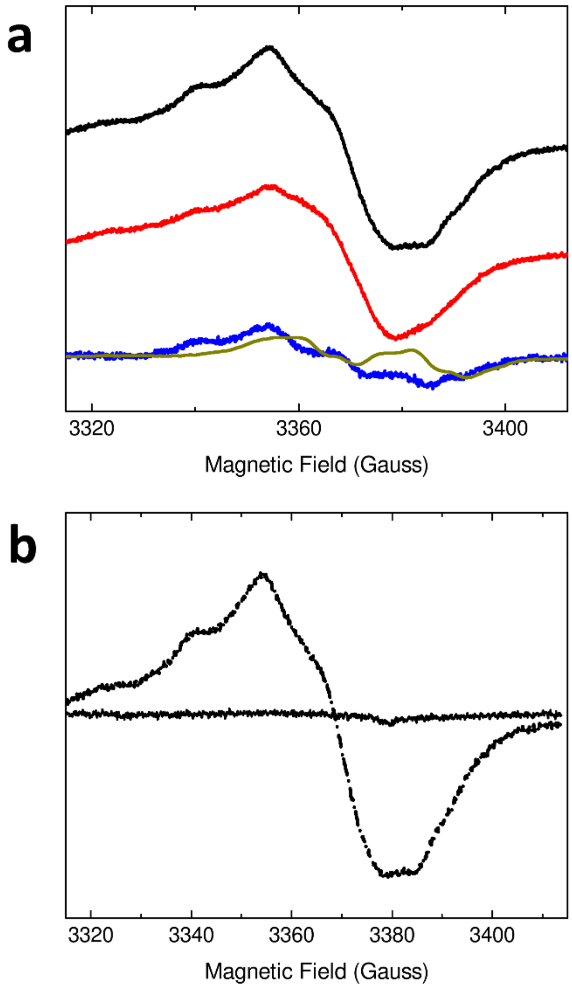

Figure S10. Radical formation during the FtMt-mediated oxidation of Fe^{2+} by H_2O_2. (**a**) Spectra of wild-type (black) and Y34F (red) FtMt anaerobically incubated with 300 µM Fe^{2+} prior to mixing with 500 µM H_2O_2 and frozen approximately 10 s after H_2O_2 addition. Blue trace shows the difference spectrum between the protein-based radical formed on the wild-type and Y34F proteins; dark yellow trace is the equivalent plot for the O_2-driven reaction. (**b**)The EPR spectrum of wild-type FtMt inhibited by pre-incubation with 60 equivalents of Zn^{2+} prior to anaerobic incubation with 300 µM of Fe^{2+} and then 500 µM H_2O_2 (solid black trace). Dashed black trace shows the equivalent spectrum recorded in the absence of Zn^{2+} demonstrating that formation of the protein-based radical is dependent on FOC activity. Samples were frozen approximately 10 s after the addition of H_2O_2. Source data are provided as a Source Data file.

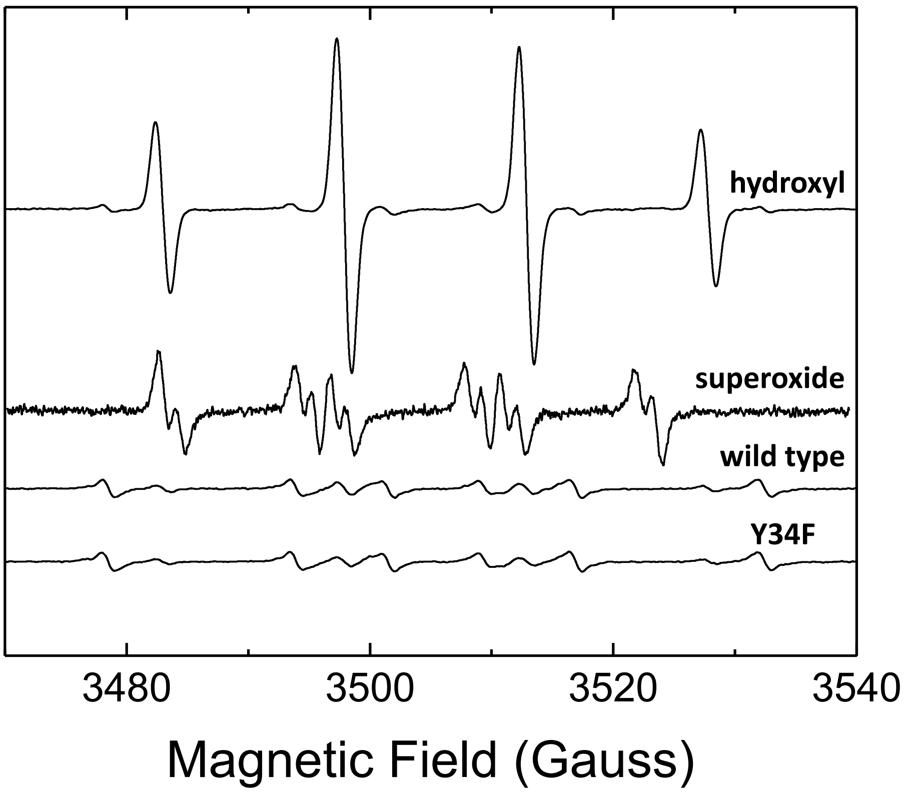

Figure S11. O_2 reduction during Fe^{2+} oxidation catalysed by FtMt. Amplex Red assays were conducted with an Fe^{2+} concentration of 9.6 μM, giving a theoretical maximum yield of 4.8 μM H_2O_2. Protein concentration was varied between 0.2 and 1.0 μM to investigate the effect of partial loading of FCs on the product of O_2 reduction. For both wild-type and variant Y34F FtMt, the concentration of H_2O_2 released, as judged by reference to a calibration plot constructed using serial dilution of a standard H_2O_2 solution, was approximately 1.1 μM, irrespective of the ratio of Fe^{2+} to protein employed. A control experiment in which 1.0 μM FtMt with di-Fe^{3+} FCs was mixed with 5 μM H_2O_2 and subjected to the Amplex Red assay also led to detection of 1.1 μM H_2O_2, suggesting that the presence of FtMt interferes with H_2O_2 detection either by direct reaction or reaction with Complex I of horse radish peroxidase, preventing oxidation of the Amplex Red dye. Radical trapping experiments during Fe^{2+} oxidation catalysed by FtMt were also performed. Shown here are EPR spectra of the hydroxyl and superoxide radical adducts of the spin trap DMPO, generated by incubating the reagent with Fe^{2+} and H_2O_2 or xanthine/xanthine oxidase respectively, together with corresponding DMPO spectra following aerobic incubation with either wild-type or variant Y34F FtMt and Fe^{2+}. The spectra following incubation with either protein are indistinguishable and demonstrate negligible formation of superoxide in either case. Thus, the data are consistent with the conclusion that peroxide is the major product of O_2 reduction for both wild-type and variant proteins. Source data are provided as a Source Data file.

Table S1 Iron oxidation catalysed by the ferroxidase centres of FtMt[a].

Fe^{2+}/FtMt	Wild-type				Y34F			
	k_{rapid} (s^{-1})	A_{rapid}	k_{slow} (s^{-1})	A_{slow}	k_{rapid} (s^{-1})	A_{rapid}	k_{slow} (s^{-1})	A_{slow}
6	0.89	0.0030	-	-	-	-	0.19	0.0006
12	1.90	0.0103	-	-	-	-	0.15	0.0040
18	2.65	0.0188	-	-	2.01	0.0025	0.19	0.0074
24	2.71	0.0244	-	-	1.65	0.0033	0.19	0.0091
30	3.08	0.0305	-	-	1.80	0.0052	0.17	0.0109
36[b]	3.22	0.0346	-	-	1.69	0.0072	0.14	0.0129
42[b]	3.68	0.0383	-	-	1.76	0.0093	0.11	0.0163
48[b]	3.94	0.0395	-	-	1.87	0.0095	0.11	0.0183
60	5.15	0.0397	0.07	0.0088	2.36	0.0114	0.09	0.0238
72	6.25	0.0390	0.07	0.0128	3.38	0.0112	0.10	0.0269
96	8.33	0.380	0.11	0.019	4.99	0.0120	0.09	0.0348

[a]Effective first order rate constants and associated amplitudes of the rapid and slow phases of FC catalysed Fe^{2+} oxidation for wild type and variant Y34F FtMt.

[b]At Fe^{2+} loadings of 36 to 48 Fe^{2+}/FtMt the wild-type protein exhibited a linear increase in absorbance at 340 nm following the rapid phase of iron oxidation. The amplitude of this increase was extremely small and did not support the extraction of an effective first order rate constant.

Supplementary references

1 Ebrahimi, K. H., Bill, E., Hagedoorn, P. L. & Hagen, W. R. The catalytic center of ferritin regulates iron storage via Fe(II)-Fe(III) displacement. *Nat. Chem. Biol.* **8**, 941-948, doi:10.1038/nchembio.1071 (2012).

2 Baaghil, S., Lewin, A., Moore, G. R. & Le Brun, N. E. Core formation in *Escherichia coli* bacterioferritin requires a functional. *Biochemistry* **42**, 14047-14056, doi:10.1021/bi035253u (2003).
