# Supplementary material for: Human mitochondrial ferritin exhibits highly unusual iron-O2 chemistry distinct from that of cytosolic ferritins
Source: Nat Commun. 2025 May 20;16:4695. doi: 10.1038/s41467-025-59463-1 (PMC12092714; doi:10.1038/s41467-025-59463-1)
Supplement: Supplementary file 2 — Reporting Summary [file 41467_2025_59463_MOESM2_ESM.pdf]

nature portfolio

Corresponding author(s): Prof Nick Le Brun

Last updated by author(s): Apr 17, 2025

Reporting Summary

Nature Portfolio wishes to improve the reproducibility of the work that we publish. This form provides structure for consistency and transparency in reporting. For further information on Nature Portfolio policies, see our Editorial Policies and the Editorial Policy Checklist.

Statistics

For all statistical analyses, confirm that the following items are present in the figure legend, table legend, main text, or Methods section.

n/a	Confirmed	
[ ]	[x]	The exact sample size (*n*) for each experimental group/condition, given as a discrete number and unit of measurement
[ ]	[x]	A statement on whether measurements were taken from distinct samples or whether the same sample was measured repeatedly
[x]	[ ]	The statistical test(s) used AND whether they are one- or two-sided<br>*Only common tests should be described solely by name; describe more complex techniques in the Methods section.*
[x]	[ ]	A description of all covariates tested
[x]	[ ]	A description of any assumptions or corrections, such as tests of normality and adjustment for multiple comparisons
[x]	[ ]	A full description of the statistical parameters including central tendency (e.g. means) or other basic estimates (e.g. regression coefficient) AND variation (e.g. standard deviation) or associated estimates of uncertainty (e.g. confidence intervals)
[x]	[ ]	For null hypothesis testing, the test statistic (e.g. *F*, *t*, *r*) with confidence intervals, effect sizes, degrees of freedom and *P* value noted<br>*Give P values as exact values whenever suitable.*
[x]	[ ]	For Bayesian analysis, information on the choice of priors and Markov chain Monte Carlo settings
[x]	[ ]	For hierarchical and complex designs, identification of the appropriate level for tests and full reporting of outcomes
[x]	[ ]	Estimates of effect sizes (e.g. Cohen's *d*, Pearson's *r*), indicating how they were calculated

Our web collection on statistics for biologists contains articles on many of the points above.

Software and code

Policy information about availability of computer code

Data collection	EPR data collection was carried out using WinEPR/XEPR (Bruker BioSpin), stopped flow data collection was with ProData SX (Applied PhotoPhysics).
Data analysis	Data analysis was performed using Origin 8 (Origin Labs).

For manuscripts utilizing custom algorithms or software that are central to the research but not yet described in published literature, software must be made available to editors and reviewers. We strongly encourage code deposition in a community repository (e.g. GitHub). See the Nature Portfolio guidelines for submitting code & software for further information.

Data

Policy information about availability of data

All manuscripts must include a data availability statement. This statement should provide the following information, where applicable:

- Accession codes, unique identifiers, or web links for publicly available datasets
- A description of any restrictions on data availability
- For clinical datasets or third party data, please ensure that the statement adheres to our policy

Relevant data are available within the paper and Supporting Information and source data files are provided.

Research involving human participants, their data, or biological material

Policy information about studies with human participants or human data. See also policy information about sex, gender (identity/presentation), and sexual orientation and race, ethnicity and racism.

Reporting on sex and gender	N/A
Reporting on race, ethnicity, or other socially relevant groupings	N/A
Population characteristics	N/A
Recruitment	N/A
Ethics oversight	N/A

Note that full information on the approval of the study protocol must also be provided in the manuscript.

Field-specific reporting

Please select the one below that is the best fit for your research. If you are not sure, read the appropriate sections before making your selection.

- [x] Life sciences
- [ ] Behavioural & social sciences
- [ ] Ecological, evolutionary & environmental sciences

For a reference copy of the document with all sections, see nature.com/documents/nr-reporting-summary-flat.pdf

Life sciences study design

All studies must disclose on these points even when the disclosure is negative.

Sample size	All absorbance kinetic experiments were repeated in triplicate, all giving very similar data. In most cases, at least two independently produced protein samples were employed, the only exceptions being the S144A and A144S variants, which were generated and characterized under time constraints in response to reviewer comments, and the experiment reported in Figure S6, which required a large quantity of protein sample. EPR slow-freeze experiments were repeated in duplicate, and a standard 10 s freeze spectrum following addition of 72 Fe2+ per protein was recorded each time any EPR experiments were run (multiple visits to the Svistunenko lab at Essex) to ensure reproducibility between different protein preparations. Rapid freeze-quench EPR experiments resulted in an entirely self-consistent set of spectra at increasing time points. This experiment was extremely time-consuming and resource-intensive due to liquid He demands and measurements were not repeated beyond the multiple time points. We confirm that all attempts at replication were successful.<br>A general statement about reproducibility is now included in the Methods section, and any exceptions to this are mentioned in the relevant figure legends.
Data exclusions	None
Replication	All absorbance kinetic experiments were repeated in triplicate, all giving very similar data. EPR slow freeze experiments were repeated in duplicate, and a standard 10 s freeze spectrum following addition of 72 Fe per protein was recorded each time any EPR experiments were run (multiple visits to the Svistunenko lab at Essex) to ensure reproducibility between different protein preparations. Rapid freeze quench EPR experiments resulted in an entirely self-consistent set of spectra at increasing time points. This experiment was extremely time-consuming and expensive due to liquid He demands and were not repeated beyond the multiple time point measurements.
Randomization	None
Blinding	None

Reporting for specific materials, systems and methods

We require information from authors about some types of materials, experimental systems and methods used in many studies. Here, indicate whether each material, system or method listed is relevant to your study. If you are not sure if a list item applies to your research, read the appropriate section before selecting a response.

Materials & experimental systems

n/a	Involved in the study
☒	☐ Antibodies
☒	☐ Eukaryotic cell lines
☒	☐ Palaeontology and archaeology
☒	☐ Animals and other organisms
☒	☐ Clinical data
☒	☐ Dual use research of concern
☒	☐ Plants

Methods

n/a	Involved in the study
☒	☐ ChIP-seq
☒	☐ Flow cytometry
☒	☐ MRI-based neuroimaging

Plants

Seed stocks	N/A
Novel plant genotypes	N/A
Authentication	N/A
